# Changes in Composition of Some Bioactive Molecules upon Inclusion of *Lacticaseibacillus paracasei* Probiotic Strains into a Standard Yogurt Starter Culture

**DOI:** 10.3390/foods12234238

**Published:** 2023-11-23

**Authors:** Konstantin V. Moiseenko, Olga A. Glazunova, Olga S. Savinova, Alexander V. Shabaev, Tatyana V. Fedorova

**Affiliations:** A. N. Bach Institute of Biochemistry, Research Center of Biotechnology, Russian Academy of Sciences, Leninsky Ave. 33/2, 119071 Moscow, Russia; mr.moiseenko@gmail.com (K.V.M.); olga.a.glas@gmail.com (O.A.G.); savinova_os@rambler.ru (O.S.S.); a.shabaev1998@gmail.com (A.V.S.)

**Keywords:** *Streptococcus thermophilus*, *Lactobacillus delbrueckii*, *Lacticaseibacillus paracasei*, yogurt, probiotics, antioxidant activity, ACE inhibition, organic acids, fatty acids, volatile organic compounds

## Abstract

Incorporation of probiotic *Lacticaseibacillus paracasei* into a standard yogurt starter culture can drastically improve its health promoting properties. However, besides being an advantage in itself, the incorporation of a new probiotic strain can significantly affect the overall composition of fermented milk. In this article, the effect of incorporation of the *L. paracasei* probiotic strains (KF1 and MA3) into several standard yogurt starter cultures (consisting of the following strains: *Streptococcus thermophilus* 16t and either *Lactobacillus delbrueckii* Lb100 or *L. delbrueckii* Lb200) was investigated. Such parameters as the degree of proteolysis, antioxidant activity, ACE-inhibitory activity, content of organic acids, profile of FAs and profile of volatile organic compounds were measured, and the influence of the starter culture composition on these parameters was described. It was demonstrated that, at least in the case of the studied strains, yogurt with *L. paracasei* had an advantage over the standard yogurt in terms of the content of acetoin, acetic acid, butyric acid and conjugated linoleic acid. Moreover, the incorporation of *L. paracasei* KF1 significantly improved the hypotensive properties of the resulting yogurt. Thus, the presented study provides insight into the bioactive molecules of probiotic yogurt and may be useful for both academia and industry in the development of new dairy-based functional products.

## 1. Introduction

Although fermented milk products have been known since the dawn of civilization, interest in this type of food is now higher than ever. Initially, fermentation was a way to preserve milk for long-term storage and fermented dairy products were considered mainly as a source of nutrition [1,2]. Over time, the shelf-life and nutritional value of fermented milk products became less important, while the fresh aroma, pleasant texture and distinctive taste of these products began to dominate consumer preferences. Nowadays, fermented dairy product have become an archetype of functional food—food that provides health benefits beyond its nutritional value upon regular consumption [3,4].

Yogurt is currently one of the most popular dairy products in the world. Although yogurt has been known for centuries as a health-promoting food, the scientific basis for its health benefits was only discovered in the early 20th century [5,6]. At this time, the Bulgarian Stamen Grigorov, who had just entered the medical faculty of the University of Geneva, discovered *Bacillus bulgaricus* (now *Lactobacillus delbrueckii* subsp. *bulgaricus*), to the consumption of which in 1909 Yulia Mechnikov attributed the longevity of the Bulgarian population [7,8]. Now, countless studies have been conducted on the health benefits of yogurt. It was demonstrated that regular consumption of yogurt can improve digestion, stimulate the immune system, and reduce the risk of cardiovascular diseases and type 2 diabetes [9,10,11].

Classically, yogurt is prepared using only milk and a starter culture consisting of *L. delbrueckii* and *Streptococcus thermophilus* [5]; however, over the past decade, many promising varieties of yogurt have been invented, including fruit or flavored yogurt, frozen yogurt, soy yogurt, and others [12]. Arguably, the most promising variety of yogurt in terms of its potential health benefits is probiotic yogurt, for which the starter culture contains additional types of lactic acid bacteria (LABs) with probiotic properties.

One of the most promising probiotic LABs that can be incorporated into the standard yogurt starter is *Lacticaseibacillus paracasei* (formerly *Lactobacillus paracasei*). *L. paracasei* is a part of the *Lacticaseibacillus casei* group that include such widely researched probiotic species as *L. casei* and *Lacticaseibacillus rhamnosus* [13,14]. Many of the isolated *L. paracasei* strains have been used as single-strain probiotics or as part of a symbiotic consortium within formulations [15,16,17]. It has been shown that regular consumption of certain strains of *L. paracasei* can strengthen the immune system, alleviate inflammation and reduce the risk of obesity [15,17,18].

Although the incorporation of *L. paracasei* into the yogurt starter is a benefit in and of itself, it should be mentioned that the fermentation of milk by a specific LAB consortium significantly changes almost all its components. The major carbohydrate present in milk, lactose, is hydrolyzed to glycose and galactose, both of which can be further fermented into organic acids [19]. The increased content of certain organic acids during fermentation leads to the unfavorable environment for the growth of pathogenic microorganisms, increases the bioavailability of micronutrients (vitamins) and promotes the absorption of calcium by the intestinal epithelium [20]. The major milk proteins (casein and whey proteins) can be break down to small molecular weight peptides (from 2 to 15 aa) with antioxidant, antihypertensive and hypocholesterolemic properties [21,22].

Usually, fermentation does not significantly affect the overall fatty acid (FA) composition of milk; however, enzymatic lipolysis during fermentation can release some flavor forming FAs [23], and more importantly, starter cultures can enrich fermented milk with specific very biologically active FAs, such as conjugated and branched chain FAs [24,25,26,27]. Both these groups of FAs possess a suppressing effect on tumor development, artery plaque formation and neurological disturbances [24,25,28,29]. Additionally, recent studies identified a very specific class of FA, branched-chain hydroxy-FA, as cell-autonomous metabolic regulators, which help to preserve whole-body glucose homeostasis and alleviate some consequences of obesity-linked type 2 diabetes [30].

All of the mentioned changes play a significant role in the transformation of fermented milk into a functional product, and the inclusion of new LABs in the starter culture can significantly affect them. Currently, there are very few publications that describe how the incorporation of *L. paracasei* into the standard yogurt starter culture influences the overall composition of the resulted product [31,32,33].

In this work, several previously described *L. paracasei* probiotic strains (KF1 and MA3) [16] were each incorporated into two different standard yogurt starter cultures, consisting of *S. thermophilus* strain 16t and either *L. delbrueckii* strain Lb100 or *L. delbrueckii* strain Lb200. In products obtained from various combinations of the mentioned strains (as well as in several single-strain fermented products) such parameters as the degree of proteolysis, antioxidant activity, Angiotensin-I-converting enzyme inhibitory (ACE-I) activity, content of organic acids, profile of FAs and profile of volatile organic compounds (VOCs) were measured, and the influence of strain composition on these parameters was described.

## 2. Materials and Methods

### 2.1. Strains and Cultivation Conditions

The strains used as classical yogurt starter culture—*S. thermophilus* 16t, *L. delbrueckii* Lb100 and *L. delbrueckii* Lb200—and probiotic strains—*L. paracasei* KF1 and *L. paracasei* MA3—were obtained from the Collection of the All-Russian Research Institute of the Dairy Industry (VNIMI, Moscow, Russia). Upon reception, all strains were stored at −80 °C in skim milk containing 20% (*v*/*v*) glycerol (i.e., glycerol-stock cultures).

To obtain the starting broth-stock cultures, the glycerol-stock cultures of *S. thermophilus* 16t and *Lactobacillus* spp. were inoculated into M17 broth medium (HiMedia Laboratories, Mumbai, India) and de Man, Rogosa and Sharpe (MRS) broth medium (HiMedia Laboratories, Mumbai, India), respectively. The incubation was carried out overnight at 37 °C for *S. thermophiles* and *L. delbrueckii* strains*,* and at 30 °C for *L. paracasei* strains.

The working culture of the LAB was obtained by inoculation of reconstituted (12%, *w*/*v*) commercial skim milk powder of the “Standard” brand (Complimilk, Slutsk cheese-making plant, Slutsk, Belarus) with the broth-stock culture (3%, *v*/*v*). Before inoculation, the milk was sterilized at 110 °C for 10 min and cooled to approximately 30 °C. Incubation was carried out overnight at the optimal growth temperature for each LAB—37 °C for *S. thermophiles* 16t and both strains of *L. delbrueckii,* and 30 °C for both strains of *L. paracasei*.

### 2.2. Milk Fermentation

The reconstituted skim milk (RSM) was pasteurized at 85 °C for 30 min and then cooled to 40 ± 2 °C. The pasteurized RSM was aseptically inoculated (1.0%, *v*/*v*) with the different combinations of working cultures presented in Table 1.

The fermentation was carried out at 37 °C until the coagulation of the milk, after which the samples were stored at 4 °C for 12 h for further analysis. All fermentations were performed in triplicate.

### 2.3. Viability of Lactic Acid Bacteria and pH Measurement

The viability of LABs was expressed as the number of colony-forming units (CFUs) per mL of culture. For *S. thermophilus* the CFUs were selectively enumerated using M17 agar (HiMedia Laboratories, Mumbai, India), and for *Lactobacillus* spp. the CFUs were selectively enumerated using MRS agar (HiMedia Laboratories, Mumbai, India) adjusted to pH 5.2. The incubation was carried out anaerobically at 37 °C for 24 and 72 h for *S. thermophilus* and *Lactobacillus* spp., respectively. Anaerobic conditions were created using Anaero Bag System 24 (HiMedia Laboratories, Mumbai, India) [34].

The pH value of fermented samples was measured with a pH-meter (Mettler Toledo, Griefensee, Switzerland) at room temperature (20 ± 2 °C).

### 2.4. Proteolytic, Antioxidant and Angiotensin-I-Converting Enzyme Inhibitory Activities

The degree of proteolysis, antioxidant activity and ACE-I activity of the samples were measured after the removal of the milk clot. The clot was separated by centrifugation at 3000× *g* for 15 min at 4 °C (Eppendorf centrifuge 5430 R, Hamburg, Germany), and the resulting supernatant was stored at −20 °C until further use. Before measurements, the supernatant was thawed at 4 °C, centrifuged at 10,000× *g* for 3 min at room temperature, and filtered through a syringe filter with a 0.45 µm hydrophilic membrane (Merk Millipore, Darmstadt, Germany).

The proteolytic activity in the resulting supernatant was measured spectrophotometrically with the 2,4,6-trinitrobenzenesulfonic acid (TNBS; Sigma-Aldrich, St. Louis, MO, USA) as substrate according to Adler-Nissen [35] with some modifications, as described in Glazunova et al. [34]. The degree of proteolysis was determined using a calibration curve constructed with L-leucine (L-Leu) in the concentration range from 0.1 to 2.0 mM, and the proteolytic activity was reported as L-Leu molar equivalents—mM (L-Leu).

The antioxidant activity in the resulting supernatant was determined by the trolox equivalent antioxidant capacity (TEAC) assay with the generation of the 2,2′-azinobis-(3-ethylbenzothiazoline-6-sulfonate radical cation (ABTS^•+^) according to Re et al. [36]. The ABTS^•+^ was prepared by incubation of a solution containing 7 mM ABTS (Sigma-Aldrich, St. Louis, MO, USA) and 2.45 mM potassium peroxodisulfate (Sigma-Aldrich, St. Louis, MO, USA) in the dark at room temperature for 12–18 h. The reaction was recorded by the decrease in OD_734_ nm for 40.5 min at 25 °C. The measurements were carried out on a Synergy 2 microplate photometer-fluorimeter (BioTek, Winooski, VT, USA). The antioxidant capacity of samples against ABTS^•+^ was reported as an amount of trolox molar equivalents—µM (TE).

The ACE activity was measured by the enzymatic cleavage of the o-Aminobenzoyl-Phe-Arg-Lys(dinitrophenyl)-Pro (Sigma-Aldrich, St. Louis, MO, USA), and the fluorescence from the cleaved substrate was recorded with a Synergy 2 microplate photometer-fluorometer (BioTek, Winooski, VT, USA). The ACE-I activity of the resulting supernatant was expressed as its half maximal inhibitory concentration (IC_50_), as described in Torkova et al. [37].

### 2.5. Organic Acid Profile Determination

Organic acid profiles of the fermented milk samples were determined by capillary electrophoresis as described in Rozhkova et al. [38]. Analysis was performed using a capillary electrophoresis system (model Kapel-105M, Lumex Ltd., St. Petersburg, Russia) capable of determining the concentration of 10 organic acids, namely oxalic, formic, tartaric, malic, citric, succinic, lactic, acetic, ascorbi, and propionic acids. The data were processed using Elforun^®^ 205 software (St. Petersburg, Russia).

### 2.6. Fatty Acid Profile Determination

The fats were extracted from the samples of fermented milk by the method proposed by Folch et al. [39]. After the extraction, the fats were hydrolyzed with simultaneous derivatization of FAs using a commercial solution of 3 M HCl in methanol (Supelco, Bellefonte, PA, USA). The separation of the derivatized FAs was performed in the regime of temperature gradient on the GC 2010 gas chromatograph (Shimadzu, Kyoto, Japan) equipped with an MDN-5 column (30 m × 0.25 mm; Bellefonte, PA, USA). Separated FAs were analyzed using a QP 2010 quadrupole mass spectrometer (Shimadzu, Kyoto, Japan). The complex qualitative mixture of FAs, PUFA-2 (Supelco, Bellefonte, PA, USA), was used as an analytical standard. The identification of the FAs was carried out as described in Moiseenko et al. [40]. The relative intensities (further relative abundances) of the FA were obtained by normalization on the total intensity of the assigned peaks. All experiments were performed in triplicate.

The FA-related nutritional indices were calculated according to Chen et al. [41]:

IA = [C12:0 + (4 × C14:0) + C16:0]/[∑MUFA + ∑PUFA](1)

HPI = [∑MUFA + ∑PUFA]/[C12:0 + (4 × C14:0) + C16:0](2)

IT = [C14:0 + C16:0 + C18:0]/[0.5 × (∑MUFA + ∑PUFA{*n*-6})] (3)

HH = [C18:1 + ∑PUFA]/[C12:0 + C14:0 + C16:0](4)

UI = 1 × (%monoenoics) + 2 × (%dienoics) + 3 × (%trienoics) + 4 × (%tetraenoics)(5)

where MUFA stands for monounsaturated fatty acids; PUFA—polyunsaturated fatty acids; IA—index of atherogenicity; HPI—health-promoting index (which is the reciprocal of IA and is mainly used in research on dairy products); IT—index of thrombogenicity; HH—hypocholesterolemic/hypercholesterolemic ratio; UI—unsaturation index.

### 2.7. Volatile Organic Compound Profile Determination

The VOC profiles of the fermented milk samples were determined as described in Moiseenko et al. [40]. In brief, the VOCs were extracted from the samples of fermented milk by solid phase microextraction using SPME Fiber Assembly Polydimethylsiloxane/Divinylbenzene (PDMS/DVB) cartridges (Supelco, Bellefonte, PA, USA). Extracted VOCs were separated using a GS 2010 gas chromatograph (Shimadzu, Kyoto, Japan) equipped with an Optima-1 column (25 m × 0.25 mm, Supelco, Bellefonte, PA, USA) and analyzed using QP 2010 quadrupole mass spectrometer (Shimadzu, Kyoto, Japan). To determine Kovats retention indices, the column was calibrated using retention index standards (Sigma, USA), containing a mixture of C8-C32 hydrocarbons. Mass detection was carried out in the range of 45–450 *m*/*z*, and the relative intensities (hereinafter referred to as relative contents) of individual VOCs were obtained by normalization to the total intensity of all peaks assigned to the VOCs. VOCs were identified using both Kovats retention indices and mass spectra. The mass spectra were compared with the reference spectra from the NIST-EPA-NIH Mass Spectral Database (NIST 11). Only peaks with an assignment reliability greater than or equal to 90% were considered valid for further analysis.

### 2.8. Statistical Analysis

All statistical comparisons between groups of samples were performed in two stages. Firstly, the one-way ANOVA omnibus *F*-Test was carried out to determine whether all compared groups were the same or if some groups were statistically significantly (*p* < 0.05) different from the others. If the presence of significantly different groups was detected, in the second stage, multiple pairwise comparisons using Tukey’s honestly significant difference (Tukey’s HSD) tests were carried out. The differences were considered significant if the *p*-values were less than 0.05 (i.e., *p* < 0.05).

## 3. Results and Discussion

### 3.1. Bacterial Growth and Medium Acidification

Data on the bacterial growth and medium acidification for the samples fermented by the different combinations of LABs are presented in Table 2. Generally, acidification capacity is an important technological factor in milk fermentation, affecting both organoleptic characteristics and storage stability of the resulting product [42]. For all studied samples, no statistically significant differences (*p* > 0.05) between the pH values at the end of fermentation were detected, and the pH value comprised 4.3 ± 0.1.

In all studied LAB consortia, *S. thermophilus* 16t was the predominant LAB. The final concentration of its cells was approximately an order of magnitude higher than that for *Lactobacillus* spp. During milk fermentation, changes in the viable cell counts of *S. thermophilus* 16t growing in monoculture was about 2.25 lg (CFU·mL^−1^), and its growth was not significantly affected by the presence of the other LABs (Table 2). Generally, CFUs of *S. thermophilus* 16t reached a value of 8.7 ± 0.1 lg (CFU·mL^−1^) at the end of fermentation; the exceptions were the Str16t + Lb200 and Str16t + Lb200 + KF1 samples, in which the viable cell counts at the end of fermentation were 8.9–9.0 lg (CFU·mL^−1^).

For the *L. delbrueckii* strains, their co-cultivation with *S. thermophilus* 16t (i.e., Str16t + Lb100 and Str16t + Lb200) resulted in a slightly higher number of viable cells (7.9 ± 0.1 lg (CFU·mL^−1^)) compared to the mono-strain fermentations (7.2 ± 0.4 lg (CFU·mL^−1^)). The incorporation of *L. paracasei* probiotic strains in yoghurt starters did not significantly affect the cell viability of *Lactobacillus* spp.; the only exception was the Str16t + Lb200 + KF1 sample in which the CFU of *Lactobacillus* spp. was significantly (*p* < 0.05) lower than in the other multi-strain fermentations, but at the same level as the fermentations with single strains of *L. delbrueckii*.

### 3.2. Proteolytic, Antioxidant and ACE-Inhibitory Activities

Proteolytic, antioxidant and ACE-I activities measured at the end of fermentation are presented in Table 3. The highest degree of proteolysis and antioxidant activity (*p* < 0.05) were detected during milk fermentation with monocultures of *L. delbrueckii*, and strain Lb100 showed higher proteolytic activity compared to strain Lb200. For the other samples, both the degree of proteolysis and antioxidant activity were the same (*p* > 0.05). The observed dependency between the degree of proteolysis and antioxidant activity of the samples substantiate previous observations about their positive correlation [43].

The values of ACE-I activity significantly varied between fermentations. It was demonstrated that the incorporation of *L. paracasei* MA3 to the starter containing *S. thermophilus* 16t and *L. delbrueckii* LB100 significantly (*p* < 0.05) increased ACE-I activity (i.e., decreased the IC_50_ value), while its incorporation into the starter containing *S. thermophilus* 16t and *L. delbrueckii* LB200 did not produce any effects. The addition of the *L. paracasei* KF1 strain significantly (*p* < 0.05) increased ACE-I activity when it was supplemented to both Str16t + Lb100 and Str16t + Lb200 starters. Interestingly, previous in silico analysis of the *L. paracasei* KF1 and *L. paracasei* MA3 proteolytic systems [16] showed that the genome of *L. paracasei* KF1 contained two genes encoding cell envelope proteinases (CEPs)—*prtP* (WP_003601280) and *prtB* (WP_003603181)—while the genome of *L. paracasei* MA3 contained three CEP genes—two *prtP* (WP_018041452 and WP_216693535) and one *prtB* (WP_216693433). Hence, the higher number of *prtP* genes can result in an increased amount of prtP enzymes, which in turn results in a decreased number of ACE-I peptides.

### 3.3. Profile of Organic Acids

Among 10 organic acids, of which the concentration was measured in the studied samples, only citric, formic, acetic and lactic acids were present in detectable concentrations (Table 4). The concentration of citric acid was the same (*p* > 0.05) in all studied samples. The concentration of formic acid in all fermented samples was the same (*p* > 0.05) and its value was three-times lower than that in the unfermented milk. The concentration of acetic acid significantly (*p* < 0.05) decreased in the samples fermented with monoculture of *S. thermophilus* 16t compared to the unfermented milk; at the same time, its concentration significantly (*p* < 0.05) increased in the samples fermented with three-strain cultures. Expectedly, lactic acid was not detected in the unfermented milk. The highest concentration of lactic acid was detected in the milk fermented with individual *L. delbrueckii* strains (Lb100 and Lb200), while the lowest concentration was in the milk fermented with a monoculture of *S. thermophilus* 16t.

The obtained data are generally consistent with those previously reported on organic acid composition of yogurts. Citric acid is always present in yogurt, since it is a natural compound of raw milk [44,45]. Typically, the concentration of citric acid in milk amounts to ~1.5 mg·mL^−1^ and does not significantly change during yogurt fermentation [46,47]. However, it should be noted that the small changes (less than 5%) in the concentration of citric acid are generally hard to detect by the current methods, since all these methods rely on an extraction procedure, the efficiency of which can vary greatly between replicates [46,47,48,49]. The presence of formic and acetic acids in unfermented milk can be explained by autoclaving. It was previously shown that both of these acids can be readily formed in thermally treated milk [50,51]. The significant increase in acetic acid concentration in the samples fermented with three-strain cultures can be explained by the presence of *L*. *paracasei*. *L*. *paracasei* is a facultative heterofermentative LAB that, in addition to lactic acid, can synthesize acetic acid, acetoin, acetaldehyde, and/or ethanol while grown on hexose sugars [13,52]. Moreover, *L*. *paracasei* can produce lactic and acetic acids from pentose sugars [53]. Additionally, it should be mentioned that *L*. *paracasei* and especially *S. thermophilus* are acetoin-producing LABs that can utilize citric acid through the diacetyl/acetoin pathway with concomitant production of acetic acid [54].

### 3.4. Profile of Total Fatty Acids

The qualitative composition and relative abundances (percentage from total) of FA in the original milk and milk fermented by different combinations of LABs are shown in Table 5. In total, 43 different FAs were detected in all studied samples. All detected FAs were subdivided into eight general groups: medium and long-chain saturated fatty acids (MCSFAs and LCSFAs), monounsaturated fatty acids (MUFAs), polyunsaturated fatty acids (PUFAs), conjugated fatty acids (CFAs), branched-chain fatty acids (BCFAs), 2-hydroxy branched-chain fatty acids (2OH-BCFAs), and oxo-saturated fatty acids (oxo-SFA).

The group of LCSFAs was the most abundant one, and all samples contained a similar (*p* > 0.05) total amount of LCSFAs (50 ± 2%). The second, third, fourth and fifth largest groups were the groups of MUFAs, MCFAs, PUFAs and BCFAs, respectively. Compared to unfermented milk, the total amount of MUFAs significantly (*p* < 0.05) decreased in the samples fermented with individual *L. delbrueckii* strains (Lb100 and Lb200) and two the LAB consortiums—Str16t + Lb200 and Str16t + Lb200 + MA3. The total amount of MCFAs significantly (*p* < 0.05) decreased in the samples fermented with *S. thermophilus* 16t either individually or as part of the consortium with other LABs and significantly (*p* < 0.05) increased in the sample fermented with *L. delbrueckii* Lb100. In contrast to MCFAs, the total amount of PUFAs significantly (*p* < 0.05) increased in the samples fermented with *S. thermophilus* 16t either individually or as part of the consortium, with the only exception being the samples fermented with Str16t + Lb100 + KF1 and Str16t + Lb200. The total amount of BCFAs was significantly (*p* < 0.05) decreased only in the sample fermented with *L. delbrueckii* Lb100 and significantly (*p* < 0.05) increased only in the sample fermented with Str16t + Lb200.

The CFAs and 2OH-BCFAs were not detected in the unfermented milk, while their abundance after fermentation varied significantly (*p* < 0.05) depending on the fermented strains. The highest CFA content (*p* < 0.05) was observed in the Str16t + Lb200 + KF1, while the highest content of 2OH-BCFAs (*p* < 0.05) was observed in the samples fermented with *L. delbrueckii* Lb100. Interestingly, it was previously shown that among dairy products fermented by eight different LAB strains, the milk fermented with *L. delbrueckii* Lb100 demonstrated significantly better hypotensive and hypocholesterolemic properties in the Spontaneously Hypertensive Rat (SHR) animal model [34]. In all the samples only one oxo-SFA, 4-oxo-pentanoic acid (4O-C5:0), was detected; its maximal abundance was observed in the sample fermented with *S. thermophilus* 16t, and its minimal abundance was observed in the samples fermented with Str16t + Lb100 + MA3, Str16t + Lb200 and Str16t + Lb200 + KF1.

**Figure 1:**
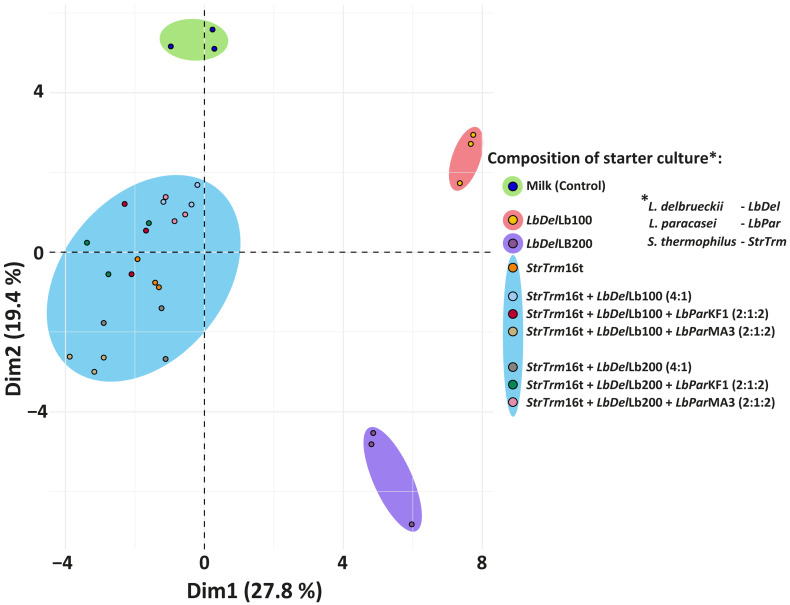
Results of principal component analysis (PCA) performed on the FA profiles of the studied samples.

Although the tabular representation of the profile of total fatty acids is quite common, it usually contains an overwhelming amount of information. For a more compact and convenient presentation of the similarities and differences in the FA composition of the studied samples, the well-known dimensionality reduction technique, PCA, was performed. As can be seen from Figure 1, all the samples depicted in the plain formed by the first two principal components (which commonly explained 47% of all observed variations in the data) agglomerated into four clusters: the first cluster was formed by the biological replicas of the unfermented milk; the second and third clusters were formed by the biological replicas of the samples fermented with the individual *L. delbrueckii* strains Lb100 and Lb200, respectively; and the fourth cluster was formed by the biological replicas of the samples fermented with *S. thermophilus* 16t either individually or as part of the consortium with other LABs. Moreover, all the samples in the fourth cluster were almost evenly spread around the samples fermented with a monoculture of *S. thermophilus* 16t.

Hence, overall FA composition in all fermentations with a mixture of LABs was primarily guided by the presence of *S. thermophilus* 16t. It was previously hypothesized that in standard yogurt consortium (consisting of *S. thermophilus* and *L. delbrueckii*) *S. thermophilus* performs the main lipolytic function, while *L. delbrueckii* performs the main proteolytic function [55]. Moreover, it was shown that the set of genes involved in long-chain fatty acid production is significantly upregulated in *S. thermophilus* during yogurt fermentation [56]. In line with that, although the total abundance of LCSFAs was unchanged in our investigation, significant (*p* < 0.05) increases in the abundances of individual LCSFAs were detected (Table 4), and all these increases were observed in the samples fermented with *S. thermophilus,* either individually or as part of the consortium with other LABs. Additionally, in our study almost all significant increases in the abundances of individual PUFAs were associated with the presence of *S. thermophilus*.

To assess the possible impact of the studied samples on the cardiovascular health in individuals who regularly consume yogurt, several well-known nutritional indices related to the FA composition of the samples were calculated (Table 6). Compared to the unfermented milk, all studied samples demonstrated significantly (*p* < 0.05) decreased IA and increased HPI, both of which convey the same information regarding the probability of developing atherosclerosis with constant use of the sample. The HPI is the inverse of the IA. While the IA is wildly used in the calculation of various diets, the HPI is most often used to assess the FA quality of dairy products [41]. The value of the IT, which reflects the probability of thrombosis development [57], was the same (*p* > 0.05) in unfermented milk and all fermented samples. The value of the HH, which reflects the probability of hypercholesterolemia development [58], was significantly increased (compared to unfermented milk) only in the sample fermented with Str16t + Lb200 + KF1. The value of the UI, which can be used to estimate the content of the high-quality PUFAs necessary to maintain the fluidity of biological membranes [59,60], was significantly (*p* < 0.05) increased in the sample fermented with Str16t + Lb200 + KF1 and significantly (*p* < 0.05) decreased in the samples fermented with individual *L. delbrueckii* strains (Lb100 and Lb200).

Regarding the general use of the FA nutritional indices for dairy products, their limitations should be mentioned. Although these indexes can be useful to some extend while comparing different dairy product with each other [34], they can be misleading while comparing dairy with other products. It is well known that dairy products contain high levels of saturated fatty acids (SFAs), the consumption of which can generally have detrimental effects on cardiovascular health [61,62]. However, recent studies suggest that milk has a neutral effect on cardiovascular outcomes, and fermented milk products have a positive or neutral effect [63,64]. In contrast, consumption of meat, which along with dairy products is one of the largest sources of SFAs in the human diet, was associated with a neutral or increased risk of cardiovascular disease [65].

### 3.5. Profile of Volatile Organic Compounds (VOCs)

The qualitative composition and relative abundances (percentage from total) of VOCs in the original milk and milk fermented by different combinations of LABs are shown in Table 7. In total, 11 different VOCs were detected in all studied samples. All detected VOCs were subdivided into four general groups: short-chain fatty acids (SCFAs), medium-chain methyl ketones (MCMKs), furan-containing compounds (FCCs), and other VOCs.

While SCFAs were not detected in the samples of unfermented milk, the group of SCFAs was the predominant one in almost all fermented samples. These FAs can be released from the milk fat through the complex biochemical transformations that mainly occur inside the LABs cells. Generally, FAs enter the cell either directly, through special transport systems, or indirectly in the form of triacylglycerides (constituting 98% of total milk fat), which are further hydrolyzed by intracellular lipases and esterases. Inside the cell, saturated FAs can be shortened due to the reaction of the *β*-oxidation cycle. Each complete cycle removes two carbon atoms from the carboxylic end of the FA. In addition, an incomplete *β*-oxidation cycle can lead to the formation of *β*-keto acids, the decarboxylation of which results in the formation of methyl ketones, which are one carbon atom less than the original FA [53,66]. Both FAs formed inside the cell and methyl ketones can subsequently be released outside the cell either by diffusion or with the participation of yet unknown transport systems [67]. Not surprisingly, the second most abundant VOC determined in our study was methyl ketones with an odd number of carbon atoms. The presence of such ketones in the original milk can be explained either by the metabolic activity of rumen microbiota or by the thermal treatment of milk before fermentation [68,69]. Similarly, the presence of 2-amylfuran and furfuryl alcohol is also a result of the milk thermal treatment during sterilization [69]. The acetoin was detected only in the samples fermented with *S. thermophilus* 16t, either individually or as part of the consortium with other LABs, and benzoic acid, only in the sample fermented with *L. delbrueckii* Lb200.

**Figure 2:**
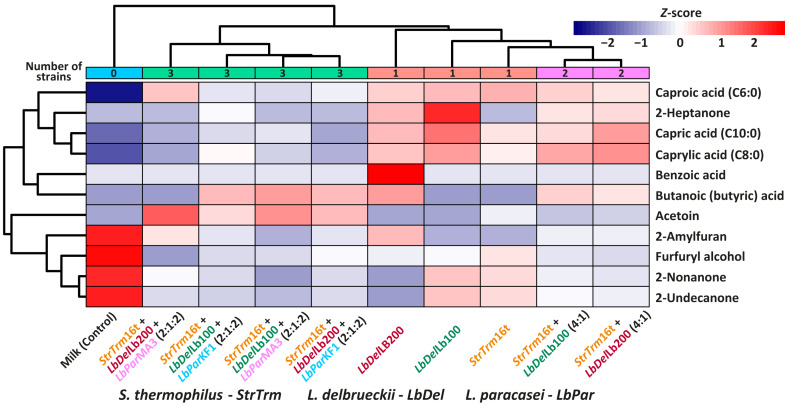
Clustered heat-map for the volatile organic compounds (VOCs) determined in the studied samples.

To investigate how the composition of the starter culture influences the VOC profile, a clustered heat-map was constructed for the volatile organic compounds (VOCs) determined in the studied samples (Figure 2). As can be seen from the clustering pattern of the columns, the VOC profile of the studied samples was primarily guided by the number of LABs in the fermented starter—the three-LABs starter cultures formed a distinct cluster, while the two-LABs starter cultures cluster was nested in a bigger cluster with a single LAB starter culture. Interestingly, the closest to the VOC profile of the cluster containing the two-LABs starter cultures was the VOC profile of the sample fermented only by *S. thermophilus* 16t.

Additionally, the heat map revealed several interesting patterns. VOC profiles of the single and two-LABs starter cultures were both characterized by the higher amount of SCFAs and MCMKs compared to the three-LABs starter cultures. At the same time, VOC profiles of the three-LABs starter cultures were significantly richer in acetoin and slightly richer in butyric acid (with the exception of Str16t + Lb200 + MA3). The acetoin production can be related to the same biochemical root as the production of acetic acid—citrate metabolism [54]. In Section 3.3, the significant increase in acetic acid concentration in the samples fermented with the three-strain cultures was especially highlighted, and the lack of citrate depletion was explained by a higher extraction error than the citrate consumption, which is required to produce the observed changes in both acetic acid and acetoin contents.

## 4. Conclusions

Summarizing all obtained results, it can be concluded that incorporation of the *L. paracasei* probiotic strains (KF1 and MA3) into the several standard yogurt starter cultures (consisting of *S. thermophilus* 16t and either *L. delbrueckii* Lb100 or *L. delbrueckii* Lb200) generally did not significantly deteriorate the composition of the resulting yogurts in terms of the biologically active molecules. Moreover, some health-related parameters were improved. The presence of either tested *L. paracasei* strains increased the content of acetic acid, butyric acid and acetoin, which not only have a positive effect on the taste of yogurt, but also have generally recognized health-beneficial properties. Also, the presence of *L. paracasei* KF1 or *L. paracasei* MA3 in the starter cultures stimulated production of conjugated linoleic acid, and the addition of the probiotic strain *L. paracasei* KF1 significantly improved the hypotensive properties of the yogurt. Thus, at least in the case of the studied combinations of LAB strains, probiotic yogurt with *L. paracasei* had an advantage over the standard yogurt, not only due to the presence of the probiotic strain itself, but also due to the improved composition of bioactive molecules.

## Figures and Tables

**Table 1:** Combination of working LAB cultures used for milk fermentation.

Strains	Ratio, *v*/*v*	Abbreviation
**One-strain fermentations**		
*L. delbrueckii* Lb100	-	-
*L. delbrueckii* Lb200	-	-
*S. thermophilus* 16t	-	-
**Two-strain fermentations**		
*S. thermophilus* 16t, *L. delbrueckii* Lb100	4:1	Str16t + Lb100
*S. thermophilus* 16t and *L. delbrueckii* Lb200	4:1	Str16t + Lb200
**Three-strain fermentations**		
*S. thermophilus* 16t, *L. delbrueckii* Lb100 and *L. paracasei* KF1	2:1:2	Str16t + Lb100 + KF1
*S. thermophilus* 16t, *L. delbrueckii* Lb100 and *L. paracasei* MA3	2:1:2	Str16t + Lb100 + MA3
*S. thermophilus* 16t, *L. delbrueckii* Lb200 and *L. paracasei* KF1	2:1:2	Str16t + Lb200 + KF1
*S. thermophilus* 16t, *L. delbrueckii* Lb200 and *L. paracasei* MA3	2:1:2	Str16t + Lb200 + MA3

**Table 2:** Bacterial growth and medium acidification in the studied samples.

Sample ^2^	pH ^1^	lg(CFU·mL^−1^) at the End of Fermentation ^1^		Δlg (CFU·mL^−1^)	
		*S. thermophilus* 16t	Total *Lactobacillus* spp.	*S. thermophilus* 16t	Total *Lactobacillus* spp.
Lb100	3.9 ^a^ (0.2)	-	7.43 ^b^ (0.13)	-	1.49
Lb200	4.1 ^a^ (0.1)	-	7.03 ^b^ (0.53)	-	1.23
Str16t	4.4^a^ (0.2)	8.86 ^a^ (0.13)	-	2.25	-
Str16t + Lb100	4.4 ^a^ (0.1)	8.87 ^a^ (0.02)	7.89 ^a^ (0.08)	2.26	1.92
Str16t + Lb100 + KF1	4.3 ^a^ (0.1)	8.68 ^a^ (0.17)	7.69 ^a^ (0.21)	2.37	1.22
Str16t + Lb100 + MA3	4.3 ^a^ (0.1)	8.66 ^a^ (0.06)	7.90 ^a^ (0.22)	2.35	1.49
Str16t + Lb200	4.3 ^a^ (0.1)	8.93 ^b^ (0.10)	7.88 ^a^ (0.07)	2.32	2.16
Str16t + Lb200 + KF1	4.3 ^a^ (0.1)	9.00 ^b^ (0.11)	7.00 ^b^ (0.30)	2.69	1.77
Str16t + Lb200 + MA3	4.3 ^a^ (0.1)	8.50 ^a^ (0.48)	7.69 ^a^ (0.13)	2.19	1.52

^1^ The data are presented as the Mean (Standard Deviation). Means within the same column with different superscripts are significantly different (*p* < 0.05). ^2^ Str16t—*Streptococcus thermophilus* 16t; Lb100—*Lactobacillus delbrueckii* Lb100; Lb200—*L. delbrueckii* Lb200; KF1—*Lacticaseibacillus paracasei* KF1; MA3—*L. paracasei* MA3.

**Table 3:** Proteolytic, antioxidant and ACE-Inhibitory activities in the studied samples.

Sample ^2^	Proteolytic Activity ^1^, mM (L-Leu)	Antioxidant Activity ^1^, µM (TE)	ACE-Inhibitory Activity IC_50_ ^1^, mg·mL^−1^
Lb100	9.93 ^a^ (0.33)	1006 ^a^ (90)	2.23 ^a^ (0.12)
Lb200	6.96 ^b^ (0.66)	888 ^b^ (75)	2.83 ^b^ (0.09)
Str16t	3.98 ^c^ (0.48)	541 ^c^ (93)	3.24 ^b^ (0.21)
Str16t + Lb100	4.99 ^c^ (0.21)	717 ^c^ (85)	3.47 ^b^ (0.32)
Str16t + Lb100 + KF1	4.96 ^c^ (0.08)	686 ^c^ (13)	1.48 ^c^ (0.17)
Str16t + Lb100 + MA3	4.80 ^c^ (0.14)	606 ^c^ (43)	2.39 ^a^ (0.21)
Str16t + Lb200	4.65 ^c^ (0.16)	646 ^c^ (77)	2.34 ^a^ (0.16)
Str16t + Lb200 + KF1	4.17 ^c^ (0.63)	625 ^c^ (18)	1.41 ^c^ (0.12)
Str16t + Lb200 + MA3	5.02 ^c^ (0.22)	646 ^c^ (25)	2.51 ^a^ (0.14)

^1^ The data are presented as the Mean (Standard Deviation). Means within the same column with different superscripts are significantly different (*p* < 0.05). ^2^ Str16t—*Streptococcus thermophilus* 16t; Lb100—*Lactobacillus delbrueckii* Lb100; Lb200—*L. delbrueckii* Lb200; KF1—*Lacticaseibacillus paracasei* KF1; MA3—*L. paracasei* MA3.

**Table 4:** Profile of organic acids in the studied samples.

Organic Acid	Relative Abundance in Sample ^1^, mg·(100 mL)^−1^									
	Milk	Lb100	Lb200	Str16t	Str16t Lb100 (4:1)	Str16t Lb100 KF1 (2:1:2)	Str16t Lb100 MA3 (2:1:2)	Str16t Lb200 (4:1)	Str16t Lb200 KF1 (2:1:2)	Str16t Lb200 MA3 (2:1:2)
Citric acid	142 ^a^ (8)	148 ^a^ (6)	145 ^a^ (8)	151 ^a^ (8)	144 ^a^ (8)	147 ^a^ (7)	154 ^a^ (5)	156 ^a^ (7)	146 ^a^ (9)	149 ^a^ (8)
Formic acid	21.6 ^a^ (2.3)	7.8 ^b^ (1.1)	6.4 ^b^ (0.8)	8.1 ^b^ (1.5)	8.4 ^b^ (2)	7.7 ^b^ (0.9)	6.7 ^b^ (1.0)	6.7 ^b^ (1.4)	7.2 ^b^ (0.8)	7.1 ^b^ (1.7)
Acetic acid	7.3 ^a^ (0.3)	7.5 ^a^ (0.2)	6.5 ^a^ (0.5)	3.1 ^b^ (0.8)	7.6 ^a^ (0.2)	8.8 ^c^ (0.3)	8.8 ^c^ (0.6)	6.5 ^a^ (1.2)	7.8 ^d^ (0.2)	7.9 ^d^ (0.1)
Lactic acid	ND	999 ^a^ (15)	865 ^b^ (21)	676 ^c^ (22)	724 ^d^ (15)	807 ^e^ (24)	836 ^e^ (14)	748 ^d^ (23)	791 ^f^ (10)	792 ^f^ (15)

^1^ The data are presented as the Mean (Standard Deviation). ND—not detected. Means within the same row with different superscripts are significantly different (*p* < 0.05). Str16t—*Streptococcus thermophilus* 16t; Lb100—*Lactobacillus delbrueckii* Lb100; Lb200—*L. delbrueckii* Lb200; KF1—*Lacticaseibacillus paracasei* KF1; MA3—*L. paracasei* MA3.

**Table 5:** Profile of total fatty acids (FAs) in the studied samples.

Fatty Acid		Relative Abundance in Samples ^1^, %									
Name	Abbreviation	Milk	Lb100	Lb200	Str16t	Str16t Lb100 (4:1)	Str16t Lb100 KF1 (2:1:2)	Str16t Lb100 MA3 (2:1:2)	Str16t Lb200 (4:1)	Str16t Lb200 KF1 (2:1:2)	Str16t Lb200 MA3 (2:1:2)
**Medium-chain saturated fatty acids (MCSFAs)**											
Hexanoic acid	C6:0	1.93 ^a^ (0.07)	3.09 ^b^ (0.15)	2.38 ^a^ (0.28)	2.09 ^a^ (0.11)	2.15 ^a^ (0.06)	2.45 ^a^ (0.38)	1.75 ^c^ (0.05)	2.12 ^a^ (0.05)	2.09 ^a^ (0.2)	2.74 ^a^ (0.28)
Heptanoic acid	C7:0	0.07 ^a^ (0.01)	0.08 ^a^ (0.01)	0.08 ^a^ (0.01)	ND	0.05 ^a^ (0.01)	0.06 ^a^ (0.01)	0.06 ^a^ (0.01)	0.07 ^a^ (0.01)	ND	0.08 ^a^ (0.01)
Octanoic acid	C8:0	3.00 ^a^ (0.48)	4.12 ^b^ (1.01)	2.41 ^a^ (0.24)	2.46 ^a^ (0.44)	2.90 ^a^ (0.40)	2.44 ^a^ (0.24)	2.23 ^a^ (0.35)	2.52 ^a^ (0.14)	2.82 ^a^ (0.21)	3.15 ^a^ (0.25)
Nonanoic acid	C9:0	0.13 ^a^ (0.01)	0.18 ^b^ (0.01)	ND	ND	0.06 ^c^ (0.02)	0.09 ^c^ (0.01)	0.05 ^c^ (0.01)	0.11 ^c^ (0.01)	0.10 ^c^ (0.01)	0.11 ^c^ (0.01)
Decanoic acid	C10:0	4.71 ^a^ (0.18)	5.31 ^a^ (1.15)	3.22 ^a^ (0.76)	2.87 ^c^ (0.17)	3.25 ^c^ (0.11)	3.10 ^c^ (0.02)	2.45 ^c^ (0.18)	2.57 ^c^ (0.53)	3.06 ^c^ (0.34)	3.54 ^c^ (0.47)
Undecanoic acid	C11:0	0.10 ^a^ (0.01)	ND	ND	ND	0.07 ^a^ (0.01)	0.05 ^a^ (0.01)	0.07 ^a^ (0.01)	ND	0.06 ^a^ (0.01)	0.07 ^a^ (0.01)
Dodecanoic acid	C12:0	4.41 ^a^ (0.72)	4.24 ^a^ (0.12)	3.61 ^a^ (0.46)	2.73 ^a^ (0.83)	3.31 ^a^ (0.20)	3.29 ^a^ (0.69)	2.68 ^a^ (0.08)	3.04 ^a^ (0.24)	2.86 ^a^ (0.23)	ND
**Total MCSFAs:**		14.3 ^a^ (1.0)	17.0 ^b^ (1.1)	11.7 ^a^ (1.6)	10.2 ^c^ (1.0)	11.80 ^c^ (0.2)	11.50 ^c^ (1.1)	9.3 ^c^ (0.5)	10.4 ^c^ (0.7)	11.0 ^c^ (0.4)	9.7 ^c^ (1.01)
**Long-chain saturated fatty acids (LCSFAs)**											
Tridecanoic acid	C13:0	0.13 ^a^ (0.01)	ND	0.17 ^a^ (0.02)	ND	0.09 ^a^ (0.01)	0.1 ^a^ (0.01)	0.08 ^a^ (0.01)	0.11 ^a^ (0.01)	ND	0.12 ^a^ (0.02)
Tetradecanoic acid	C14:0	10.13 ^a^ (0.5)	5.77 ^b^ (1.01)	5.37 ^b^ (1.08)	7.89 ^b^ (0.70)	8.44 ^a^ (0.86)	8.51 ^a^ (0.93)	7.39 ^b^ (0.51)	7.28 ^b^ (1.86)	7.09 ^b^ (1.36)	7.98 ^a^ (1.48)
Pentadecanoic acid	C15:0	1.53 ^a^ (0.31)	1.41 ^a^ (0.06)	2.55 ^b^ (0.26)	1.48 ^a^ (0.15)	1.46 ^a^ (0.11)	1.63 ^a^ (0.25)	1.54 ^a^ (0.25)	1.74 ^a^ (0.3)	1.47 ^a^ (0.08)	1.52 ^a^ (0.02)
Hexadecanoic acid	C16:0	22.37 ^a^ (2.11)	24.35 ^a^ (3.94)	23.05 ^a^ (6.04)	23.65 ^a^ (0.83)	24.57 ^a^ (1.01)	22.18 ^a^ (3.01)	23.10 ^a^ (0.96)	25.46 ^a^ (1.40)	22.05 ^a^ (0.74)	26.81 ^a^ (0.94)
Heptadecanoic acid	C17:0	0.55 ^a^ (0.08)	0.67 ^b^ (0.03)	ND	0.86 ^b^ (0.24)	0.66 ^a^ (0.02)	0.7 ^b^ (0.17)	0.96 ^b^ (0.28)	1.03 ^b^ (0.20)	0.85 ^b^ (0.04)	0.79 ^a^ (0.10)
Octadecanoic acid	C18:0	10.06 ^a^ (0.25)	11.03 ^a^ (0.39)	15.6 ^b^ (3.35)	12.65 ^b^ (0.61)	9.79 ^a^ (2.56)	11.74 ^b^ (0.24)	12.86 ^b^ (0.87)	11.92 ^a^ (1.56)	11.93 ^b^ (0.59)	11.95 ^b^ (0.48)
Eicosanoic acid	C20:0	0.38 ^a^ (0.13)	0.43 ^a^ (0.11)	0.50 ^b^ (0.02)	0.75 (0.05)	0.36 ^a^ (0.05)	0.43 ^b^ (0.03)	0.41 ^b^ (0.03)	0.62 (0.05)	0.46 ^b^ (0.01)	0.44 ^b^ (0.04)
Docosanoic acid	C22:0	1.07 ^a^ (0.04)	1.26 ^a^ (0.1)	1.35 ^a^ (0.14)	1.56 ^b^ (0.06)	1.48 ^b^ (0.04)	1.6 ^b^ (0.06)	1.73 ^b^ (0.06)	1.43 ^b^ (0.25)	1.31 ^b^ (0.15)	1.41 ^b^ (0.21)
Tricosanoic acid	C23:0	1.19 ^a^ (0.07)	1.39 ^a^ (0.16)	1.39 ^a^ (0.29)	1.87 ^b^ (0.2)	1.56 ^a^ (0.41)	1.79 ^b^ (0.46)	1.65 ^a^ (0.15)	1.78 ^b^ (0.27)	1.58 ^a^ (0.23)	1.49 ^a^ (0.11)
Tetracosanoic acid	C24:0	0.72 ^a^ (0.11)	0.79 ^a^ (0.04)	1.06 ^a^ (0.2)	1.19 ^b^ (0.13)	1.08 ^a^ (0.04)	1.27 ^b^ (0.09)	1.46 ^b^ (0.03)	1.12 ^a^ (0.14)	0.96 ^a^ (0.12)	1.02 ^a^ (0.09)
**Total LCSFAs:**		48.11 ^a^ (2.33)	47.11 ^a^ (3.11)	51.04 ^a^ (3.33)	51.89 ^a^ (1.51)	49.50 ^a^ (2.23)	49.94 ^a^ (1.57)	51.17 ^a^ (1.38)	52.48 ^a^ (1.23)	47.69 ^a^ (1.74)	53.52 ^a^ (0.93)
**Monounsaturated fatty acids (MUFAs)**											
4-Decenoic acid	C10:1 (n-6)	0.92 ^a^ (0.03)	1.05 ^a^ (0.1)	0.61 ^b^ (0.04)	0.62 ^b^ (0.06)	0.63 ^b^ (0.13)	0.64 ^b^ (0.08)	0.54 ^b^ (0.05)	0.55 ^b^ (0.04)	0.67 ^b^ (0.03)	0.69 ^b^ (0.05)
5-Dodecenoic acid	C12:1 (n-7)	ND	0.19 ^a^ (0.02)	ND	ND	0.14 ^a^ (0.01)	ND	ND	0.15 ^a^ (0.02)	ND	0.16 ^a^ (0.01)
9-Tetradecenoic acid	C14:1 (n-5)	1.01 ^a^ (0.08)	0.24 ^b^ (0.01)	0.48 ^b^ (0.04)	0.38 ^b^ (0.08)	0.41 ^b^ (0.07)	0.46 ^b^ (0.04)	0.37 ^b^ (0.05)	0.50 ^b^ (0.01)	0.44 ^b^ (0.02)	0.54 ^b^ (0.07)
9-Hexadecenoic acid	C16:1 (n-7)	1.9 ^a^ (0.08)	0.66 ^b^ (0.12)	0.96 ^b^ (0.11)	1.33 ^b^ (0.15)	1.36 ^b^ (0.09)	1.51 ^b^ (0.08)	1.42 ^b^ (0.26)	1.44 ^b^ (0.11)	1.56 ^b^ (0.22)	1.41 ^b^ (0.1)
10-Heptadecenoic acid	C17:1 (n-7)	0.28 ^a^ (0.06)	ND	ND	0.27 ^a^ (0.02)	0.26 ^a^ (0.04)	0.28 ^a^ (0.01)	0.30 ^a^ (0.04)	0.28 ^a^ (0.01)	0.24 ^a^ (0.04)	0.29 ^a^ (0.02)
12-Octadecenoic acid	C18:1 (n-6)	1.79 ^a^ (0.06)	1.82 ^a^ (0.11)	2.34 ^b^ (0.11)	1.99 ^a^ (0.13)	2.09 ^a^ (0.28)	2.05 ^a^ (0.22)	2.41 ^b^ (0.27)	2.43 ^b^ (0.28)	2.25 ^b^ (0.17)	2.08 ^a^ (0.14)
9-Octadecenoic acid	C18:1 (n-9)	22.16 ^a^ (2.68)	18.85 ^a^ (0.29)	19.14 ^a^ (0.61)	20.96 ^a^ (2.92)	22.07 ^a^ (2.17)	21.52 ^a^ (1.09)	21.04 ^a^ (0.53)	19.31 ^a^ (0.86)	22.39 ^a^ (2.64)	18.91 ^a^ (1.5)
6-Octadecenoic acid	C18:1 (n-12)	1.52 ^a^ (0.15)	1.37 ^a^ (0.13)	1.91 ^b^ (0.3)	2.04 ^b^ (0.26)	1.76 ^b^ (0.04)	1.62 ^a^ (0.34)	2.68 ^b^ (0.35)	2.29 ^b^ (0.03)	2.17 ^b^ (0.3)	1.74 ^a^ (0.26)
10-Nonadecenoic acid	C19:1 (n-9)	ND	0.38 ^b^ (0.04)	0.58 (0.09)	0.11 ^a^ (0.01)	0.17 ^a^ (0.01)	0.18 ^a^ (0.01)	ND	ND	ND	0.15 ^a^ (0.02)
11-Eicosenoic acid	C20:1 (n-9)	0.06 ^a^ (0.01)	ND	ND	0.64 ^b^ (0.07)	0.22 ^c^ (0.03)	0.57 ^b^ (0.02)	0.58 ^b^ (0.09)	0.53 ^b^ (0.02)	0.54 ^b^ (0.06)	0.67 ^b^ (0.1)
**Total MUFAs:**		29.64 ^a^ (2.52)	24.55 ^b^ (0.36)	26.03 ^b^ (0.52)	28.35 ^a^ (2.67)	29.11 ^a^ (2.39)	28.83 ^a^ (0.97)	29.34 ^a^ (0.74)	27.48 ^b^ (0.85)	30.27 ^a^ (1.96)	26.65 ^b^ (1.26)
**Polyunsaturated fatty acids (PUFAs)**											
9,12-Octadecadienoic acid	C18:2 (n-6)	4.93 ^a^ (0.9)	4.18 ^a^ (0.19)	4.38 ^a^ (0.82)	5.78 ^b^ (0.2)	5.62 ^b^ (0.7)	5.04 ^a^ (0.6)	5.87 ^b^ (0.65)	5.25 ^a^ (1.06)	6.45 ^b^ (0.42)	5.51 ^b^ (0.31)
8,11,14-Eicosatrienoic acid	C20:3 (n-6)	0.36 ^a^ (0.01)	0.36 ^a^ (0.01)	0.43 ^b^ (0.03)	0.46 ^b^ (0.01)	0.45 ^b^ (0.05)	0.49 ^b^ (0.01)	0.42 ^b^ (0.06)	0.44 ^b^ (0.03)	0.51 ^b^ (0.13)	0.47 ^b^ (0.11)
5,8,11,14-Eicosatetraenoic acid	C20:4 (n-6)	0.51 ^a^ (0.06)	0.38 ^b^ (0.02)	0.53 ^a^ (0.11)	0.68 (0.08)	0.64 (0.03)	0.71 (0.1)	0.75 (0.12)	0.55 ^a^ (0.07)	0.64 ^a^ (0.12)	0.59 ^a^ (0.04)
7,10,13,16-Docosatetraenoic acid	C22:4 (n-6)	0.27 ^a^ (0.04)	0.40 ^b^ (0.03)	ND	0.36 ^b^ (0.01)	0.37 ^b^ (0.05)	0.48 ^b^ (0.06)	0.37 ^b^ (0.02)	0.33 ^b^ (0.04)	0.45 ^b^ (0.07)	0.38 ^b^ (0.03)
7,10,13,16,19-Docosapentaenoic acid	C22:5 (n-3)	0.06 ^a^ (0.01)	ND	ND	ND	0.09 ^b^ (0.01)	0.09 ^b^ (0.01)	ND	ND	ND	ND
**Total PUFAs:**		6.13 ^a^ (0.84)	5.31 ^a^ (0.21)	5.34 ^a^ (0.86)	7.29 ^b^ (0.23)	7.17 ^b^ (0.63)	6.81 ^a^ (0.46)	7.41 ^b^ (0.76)	6.56 ^a^ (0.99)	8.05 ^b^ (0.26)	6.94 ^b^ (0.29)
**Conjugated fatty acids (CFAs)**											
10-trans,12-cis-Octadecadienoic acid	10trans,12cis-C18:2	ND	ND	ND	ND	ND	0.58 ^a^ (0.05)	0.38 ^b^ (0.06)	0.42 ^b^ (0.05)	0.66 ^a^ (0.04)	0.43 ^b^ (0.1)
9-cis,11-trans-Octadecadienoic acid	9cis,11trans-C18:2	ND	ND	0.51 ^a^ (0.1)	ND	ND	0.15 ^b^ (0.01)	0.35 ^c^ (0.02)	0.31 ^c^ (0.02)	0.23 ^d^ (0.06)	0.19 ^d^ (0.02)
**Total CFAs:**		ND	ND	0.51 ^a^ (0.1)	ND	ND	0.72 ^b^ (0.05)	0.73 ^b^ (0.05)	0.73 ^b^ (0.04)	0.89 ^c^ (0.08)	0.62 ^a^ (0.1)
**Branched-chain fatty acids (BCFA)**											
Tridecanoic acid, 12-methyl-	12MeC13:0 (iso-C14:0)	0.11 ^a^ (0.02)	ND	ND	ND	0.10 ^a^ (0.01)	0.11 ^a^ (0.02)	0.09 ^a^ (0.01)	0.10 ^a^ (0.02)	0.09 ^a^ (0)	0.11 ^a^ (0)
Tetradecanoic acid, 13-methyl-	13MeC14:0 (iso-C15:0)	0.19 ^a^ (0.05)	0.09 ^b^ (0.02)	ND	0.18 ^a^ (0.01)	0.15 ^a^ (0.01)	0.16 ^a^ (0.02)	0.16 ^a^ (0.02)	0.19 ^a^ (0.02)	0.15 ^a^ (0.01)	0.19 ^a^ (0.02)
Tetradecanoic acid, 9-methyl-	9MeC14:0	0.46 ^a^ (0.01)	0.21 ^b^ (0.04)	0.28 ^b^ (0.01)	0.38 ^b^ (0.01)	0.32 ^b^ (0.03)	0.37 ^b^ (0.05)	0.32 ^b^ (0.02)	0.39 ^b^ (0.04)	0.39 ^b^ (0.04)	0.41 ^a^ (0.05)
Pentadecanoic acid, 14-methyl-	14MeC15:0 (iso-C16:0)	0.23 ^a^ (0.04)	0.27 ^a^ (0.03)	0.26 ^a^ (0.04)	0.22 ^a^ (0.04)	0.27 ^a^ (0)	0.25 ^a^ (0.02)	0.28 ^a^ (0.03)	0.30 ^a^ (0.02)	0.26 ^a^ (0.03)	0.27 ^a^ (0.02)
Hexadecanoic acid, 15-methyl-	15MeC16:0 (iso-C17:0)	0.22 ^a^ (0.01)	0.23 ^a^ (0.01)	0.34 ^b^ (0.02)	0.23 ^a^ (0.03)	0.29 ^b^ (0.04)	0.31 ^b^ (0.05)	0.37 ^b^ (0.02)	0.40 ^b^ (0.06)	0.32 ^b^ (0.02)	0.28 ^a^ (0.04)
Hexadecanoic acid, 14-methyl-	14MeC16:0 (anteiso-C17:0)	0.36 ^a^ (0.09)	0.38 ^a^ (0.03)	0.55 ^b^ (0.13)	0.43 ^a^ (0.1)	0.44 ^a^ (0.05)	0.36 ^a^ (0.1)	0.42 ^a^ (0.09)	0.48 ^b^ (0.03)	0.38 ^a^ (0.06)	0.38 ^a^ (0.04)
**Total BCFA:**		1.57 ^a^ (0.03)	1.17 ^b^ (0.13)	1.43 ^a^ (0.16)	1.44 ^a^ (0.1)	1.58 ^a^ (0.1)	1.55 ^a^ (0.13)	1.64 ^a^ (0.09)	1.84 ^c^ (0.11)	1.59 ^a^ (0.02)	1.63 ^a^ (0.02)
**2-Hydroxy branched-chain fatty acids (2OH-BCFAs)**											
Pentanoic acid, 2-hydroxy-4-methyl-	2OH-4MeC5:0 (2OH-iso-C6:0)	ND	3.38 ^a^ (0.62)	3.31 ^a^ (0.55)	0.19 ^b^ (0.04)	0.26 ^c^ (0.03)	0.23 ^b^ (0.03)	0.17 ^b^ (0.01)	0.19 ^b^ (0.02)	0.21 ^b^ (0.04)	0.32 ^d^ (0.01)
Pentanoic acid, 2-hydroxy-3-methyl-	2OH-3MeC5:0 (2OH-anteiso-C6:0)	ND	1.15 ^a^ (0.03)	0.49 ^b^ (0.06)	0.23 ^c^ (0.07)	0.23 ^c^ (0.02)	0.17 ^c^ (0.03)	0.16 ^d^ (0.01)	0.17 ^d^ (0.02)	0.20 ^c^ (0.01)	0.30 ^f^ (0.03)
**Total 2OH-BCFAs:**		ND	4.52 ^a^ (0.64)	3.80 ^a^ (0.61)	0.42 ^b^ (0.11)	0.49 ^c^ (0.03)	0.4 ^b^ (0.03)	0.32 ^c^ (0.01)	0.37 ^c^ (0.02)	0.41 ^b^ (0.03)	0.62 ^d^ (0.05)
**oxo-Saturated fatty acids (oxo-SFAs)**											
Pentanoic acid, 4-oxo-	4O-C5:0	0.20 ^a^ (0.02)	0.32 ^b^ (0.02)	0.16 ^a^ (0.01)	0.47 ^c^ (0.03)	0.36 ^b^ (0.03)	0.27 ^b^ (0.03)	0.12 ^d^ (0.01)	0.12 ^d^ (0.01)	0.15 ^d^ (0.01)	0.33 ^b^ (0.06)
**Total oxo-SFA** **s** **:**		0.20 ^a^ (0.02)	0.32 ^b^ (0.02)	0.16 ^a^ (0.01)	0.47 ^c^ (0.03)	0.36 ^b^ (0.03)	0.27 ^b^ (0.03)	0.12 ^d^ (0.01)	0.12 ^d^ (0.01)	0.15 ^d^ (0.01)	0.33 ^b^ (0.06)

^1^ The data are presented as the Mean (Standard Deviation). Means within the same row with different superscripts are significantly different (*p* < 0.05). Str16t—*Streptococcus thermophilus* 16t; Lb100—*Lactobacillus delbrueckii* Lb100; Lb200—*L. delbrueckii* Lb200; KF1—*Lacticaseibacillus paracasei* KF1; MA3—*L. paracasei* MA3.

**Table 6:** Fatty acid (FA)-related nutritional indices of the studied samples.

Index Information		Index Value for the Samples ^1^, Arbitrary Units									
Name	GENERAL Interpretation	Milk	Lb100	Lb200	Str16t	Str16t Lb100 (4:1)	Str16t Lb100 KF1 (2:1:2)	Str16t Lb100 MA3 (2:1:2)	Str16t Lb200 (4:1)	Str16t Lb200 KF1 (2:1:2)	Str16t Lb200 MA3 (2:1:2)
Index of atherogenicity (IA)	The lower, the better	1.89 ^a^ (0.05)	1.73 ^b^ (0.01)	1.54 ^b^ (0.36)	1.64 ^b^ (0.24)	1.7 ^b^ (0.07)	1.67 ^b^ (0.03)	1.51 ^b^ (0.08)	1.69 ^b^ (0.15)	1.4 ^b^ (0.22)	1.75 ^b^ (0.15)
Health-promoting index (HPI)	The higher, the better	0.53 ^a^ (0.01)	0.58 ^b^ (0.01)	0.68 ^b^ (0.18)	0.62 ^b^ (0.09)	0.59 ^b^ (0.02)	0.61 ^b^ (0.01)	0.66 ^b^ (0.04)	0.59 ^b^ (0.05)	0.73 ^b^ (0.12)	0.57 ^b^ (0.05)
Index of thrombogenicity (IT)	The lower, the better	2.36 ^a^ (0.23)	2.76 ^a^ (0.25)	2.81 ^a^ (0.32)	2.49 ^a^ (0.25)	2.34 ^a^ (0.27)	2.35 ^a^ (0.16)	2.36 ^a^ (0.16)	2.63 ^a^ (0.22)	2.15 ^a^ (0.18)	2.79 ^a^ (0.14)
Hypo/hyper-cholesterolemic ratio (HH)	The higher, the better	0.77 ^a^ (0.09)	0.71 ^a^ (0.16)	0.80 ^a^ (0.23)	0.83 ^a^ (0.14)	0.81 ^a^ (0.07)	0.84 ^a^ (0.06)	0.86 ^a^ (0.04)	0.72 ^a^ (0.15)	0.96 ^b^ (0.08)	0.74 ^a^ (0.12)
Unsaturation index (UI)	The higher, the better	44.02 ^a^ (1.84)	37.09 ^b^ (0.41)	38.19 ^b^ (1.94)	45.46 ^a^ (2.94)	46.19 ^a^ (2.15)	45.61 ^a^ (0.87)	46.82 ^a^ (1.99)	42.8 ^a^ (2.69)	49.04 ^c^ (1.9)	42.94 ^a^ (0.98)

^1^ The data are presented as the Mean (Standard Deviation). Means within the same row with different superscripts are significantly different (*p* < 0.05). Str16t—*Streptococcus thermophilus* 16t; Lb100—*Lactobacillus delbrueckii* Lb100; Lb200—*L. delbrueckii* Lb200; KF1—*Lacticaseibacillus paracasei* KF1; MA3—*L. paracasei* MA3.

**Table 7:** Profile of volatile organic compounds (VOCs) in the studied samples.

Volatile Compound	Relative Abundance in Sample, %									
	Milk	Lb100	Lb200	Str16t	Str16t Lb100 (4:1)	Str16t Lb100 KF1 (2:1:2)	Str16t Lb100 MA3 (2:1:2)	Str16t Lb200 (4:1)	Str16t Lb200 KF1 (2:1:2)	Str16t Lb200 MA3 (2:1:2)
**Short-chain fatty acids (SCFAs)**										
Butanoic (butyric) acid (C4:0)	ND	ND	19	ND	14	16	19	12	16	ND
Caproic acid (C6:0)	ND	32	30	34	30	22	21	28	23	32
Caprylic acid (C8:0)	ND	23	19	16	22	16	11	24	8	7.3
Capric acid (C10:0)	ND	7.1	5.4	4.6	4.4	2.7	2.9	6.1	1.2	1.6
Total SCFAs:	ND	62	73	55	70	57	54	70	48	41
**Medium-chain methyl ketones (MCMKs)**										
2-Heptanone	ND	8.5	4	ND	3.3	2.5	ND	3.3	ND	ND
2-Nonanone	24	12	ND	10	5.8	4.7	ND	5.5	4.3	7.1
2-Undecanone	7	3.4	ND	3.4	2	1.2	0.7	1.8	1.3	1.5
Total MCMKs:	31	24	4	13	11	8.4	0.7	11	5.6	8.6
**Furan-containing compounds (FCCs)**										
2-Amylfuran	14	ND	6.5	ND	3	2.5	ND	2.8	2.2	4.5
Furfuryl alcohol	55	14	13	19	11	9.4	8.4	9.6	15	ND
Total FCCs:	69	14	20	19	14	12	8	12	17	4
**Other VOCs**										
Acetoin	ND	ND	ND	13	4.5	23	37	6.9	29	46
Benzoic acid	ND	ND	3.1	ND	ND	ND	ND	ND	ND	ND
Total Other VOCs:	ND	ND	3.1	13	4.5	23	37	6.9	29	46

The measurement error did not exceed the one unit of the least significant digit. ND—not detected. Str16t—*Streptococcus thermophilus* 16t; Lb100—*Lactobacillus delbrueckii* Lb100; Lb200—*L. delbrueckii* Lb200; KF1—*Lacticaseibacillus paracasei* KF1; MA3—*L. paracasei* MA3.

## Data Availability

All data are contained within the article.
